# Ruxolitinib for Therapy of Graft-versus-Host Disease

**DOI:** 10.1155/2019/8163780

**Published:** 2019-03-06

**Authors:** Thomas Neumann, Laila Schneidewind, Martin Weigel, Andrzej Plis, Rem Vaizian, Christian A. Schmidt, William Krüger

**Affiliations:** University Medicine Greifswald, Internal Medicine C, Hematology and Oncology, Stem Cell Transplantation and Palliative Care, Ferdinand-Sauerbruch-Strasse, 17475 Greifswald, Germany

## Abstract

**Objective:**

Steroid-resistant graft-versus-host disease (GvHD) is a major challenge after allogeneic stem cell transplantation and associated with significant morbidity and mortality. There is no therapeutic standard defined beyond calcineurin inhibitors (CNI) and steroids. Furthermore, some patients may have contraindications against CNI or high-dose steroids. Efficacy of ruxolitinib against GvHD has been described recently.

**Methods:**

Ruxolitinib was used for treatment of acute or chronic GvHD in eight patients. The patients either needed intensification of therapy or had contraindications against use of CNI or high-dose steroids.

**Results:**

Supplementation of therapy in acute GvHD with severe diarrhea with ruxolitinib was unsuccessful. All these patients died from acute GvHD. Introduction of ruxolitinib into therapy and relapse prophylaxis in other patients was successful in 4/4 cases (CR=3, PR=1). Indications for ruxolitinib were contraindications against CNI due to aHUS in two cases and the need for steroid sparing in two other cases. None of these patients suffered from diarrhea at the initiation of ruxolitinib.

**Conclusion:**

Ruxolitinib was effective for therapy of acute and chronic GvHD in higher lines in patients without severe diarrhea. Ruxolitinib could replace successfully CNI and high-dose steroids. Further investigations are necessary to define the position of ruxolitinib in GvHD-therapy.

## 1. Introduction

Still five decades after introduction of allogeneic transplantation of haemopoietic stem cells graft-versus-host disease (GvHD) is a leading cause for morbidity and mortality after transplantation. The incidence of GvHD may be as high as >50% [1]. The standard first line therapy of acute and chronic GvHD is the administration of steroids in conjunction with calcineurin inhibitors (CNI) [2]. However, prolonged and/or intensive steroid exposition is associated with a variety of side effects such as increased infection rates, myelopathy, and atrophy of the skin. Beyond the first line therapy, there is no standard defined so far [3]. Steroid-resistant acute GvHD is difficult to treat and associated with a high mortality. Common drugs and measures used in this situation are mycophenolate mofetil, extra corporal photopheresis, additional topical steroids, pentostatin, and antibodies such as alemtuzumab and antithymocyte globulin; however, success rates of these approaches are moderate [3]. Furthermore, intensive immunosuppression may abolish graft-versus-malignancy effects.

Ruxolitinib is an inhibitor of Janus kinases 1/2 developed for therapy of myeloproliferative diseases. Spoerl et al. described in 2014 the reduced proliferation of t-effector cells and a suppression of proinflammatory cytokine production and positive effects of ruxolitinib in experimental murine GvHD [4]. Zeiser et al. published one year later their landmark paper of successful therapy of human GvHD with ruxolitinib [5].

Here, we describe our experience with ruxolitinib in therapy of acute and chronic graft-versus-host disease. The reasons for the choice of the JAK-2 inhibitor were an unsatisfying response of GvHD to preceding therapy lines, the necessity to spare steroids, or a contraindication against CNI.

## 2. Patients and Methods

Eight patients received ruxolitinib for therapy of acute or chronic GvHD. One patient (12,5%) was female and the other patients (n=7, 87,5%) were male. The indications for allogeneic SCT (alloSCT) were acute myeloid leukaemia in three cases (37,5%) and small cell lymphocytic lymphoma, chronic lymphocytic leukaemia, multiple myeloma, follicular lymphoma, and osteomyelofibrosis in one case (12,5%) each. The median age was 57 years (range 36-68 years) at alloSCT. Seven (87,5%) patients were grafted from unrelated donors (10/10 match) and the woman suffering from multiple myeloma received a graft from her HLA-identical sibling (Table 1). In all cases G-CSF mobilized peripheral stem cells were used for transplantation. Cyclosporine-A (CSP) in conjunction with short-course methotrexate or mycophenolate mofetil (MMF) was given for GvHD prophylaxis and all patients received antibody mediated in vivo T-cell depletion within conditioning. Engraftment took place within normal interval.

Diagnosis and grading of acute and chronic GvHD followed published criteria [6, 7]. Histological confirmation was attempted whenever reasonable and possible. Graft-versus-host disease was diagnosed at day +87 (median, range +30-+224) after allogeneic transplantation. Prior to initiation of ruxolitinib therapy, six patients suffered from grade IV (n=4) or grade III (n=2) acute GvHD with gut involvement. Diarrhea was controlled by standard therapy of GvHD in 2 cases (#1, #7).

Two patients were admitted for primary chronic GvHD (#3) and for chronic GvHD as part of an overlap-syndrome (#6). Both had severe pulmonary involvement. Therapy of acute and chronic GvHD followed international standards with steroids as backbone [3, 8]. In addition, mycophenolate mofetil, extracorporal photopheresis, and mesenchymal stem cells were given.

Ruxolitinib was administered in higher therapy lines in doses between 5mg and 15mg according the publication from Zeiser et al. [5]. In general, ruxolitinib was initiated with 5mg or 10mg and doses were increased depending on patient's tolerance. The main parameter which encouraged us for a dose increase was a stable count of leukocytes and platelets under therapy. For the dosing of ruxolitinib in GvHD no standard exists so far. In the case of acute GvHD associated diarrhea, the tablets were finely grounded in a mortar to improve the resorption.

## 3. Results

An overview about patients, characteristics of GvHD, treatment, response, and outcome is shown in Tables 1 and 2. In general, at initiation of ruxolitinib four patients (#2, #4, #5, and #8) suffered from steroid-refractory acute GvHD (grade IV), three patients (#1, #6, and #7) from an overlap-syndrome after acute GvHD (grade III), and one patient (#3) from primary chronic GvHD. GvHD was diagnosed between day +30 and day + 224 (median: d +87) after allogeneic transplantation. The patients with steroid-refractory acute GvHD received ruxolitinib in higher (4^th^ to 5^th^) therapy lines in conjunction with other immunosuppressive agents, e.g., cyclosporine-A, steroids, and mycophenolate mofetil, to achieve control about uncontrolled acute GvHD with severe diarrhea. In these patients the supplementation of GvHD-therapy with ruxolitinib did not led to improvement and all of these patients (#2, #4, #5, and #8) have died from uncontrolled GvHD between day +133 and +209 after allogeneic SCT (Table 1).

The patients with overlap-syndrome (#1, #6, and #7) or with primary chronic GvHD (#3) responded within 14 days to treatment with ruxolitinib. However, no patient suffered from active GvHD of the gut with diarrhea when the JAK-2 inhibitor was started. GvHD of skin, liver, mucosa, and even severe pulmonary symptoms in patients #2 and #6 responded favorable to supplementation of GvHD-therapy with ruxolitinib. In patient #1 ruxolitinib was given for replacement of CNI due to atypical HUS and for steroid sparing. Ruxolitinib has been initially combined with steroids and MMF. Signs of GvHD regressed completely under continued monotherapy with ruxolitinib and even this therapy could be discontinued on day +804 at a patient's Karnofski score of 90%. Patient #3: chronic GvHD, pulmonary manifestation included, improved strikingly under combined treatment including ruxolitinib. Unfortunately, the patient died from pulmonary infection under double immunosuppression with MMF and ruxolitinib. Patient #6 developed an Aspergillosis under conventional treatment of cGvHD. Extracorporal photopheresis and steroids had to be discontinued and pulmonary GvHD exacerbated. In addition, the patient developed severe side effects of steroids. The combination of ruxolitinib with MMF and steroids in decreasing doses led even here to a clear improvement of pulmonary GvHD and nightly artificial respiration could be terminated. The patient is alive on day +1392 with a Karnofski score of 60%. Patient #7 developed stage III acute GvHD from day +30 after alloSCT. Steroids in conjunction with cyclosporine-A and MMF improved the GvHD and terminated diarrhea. However, GvHD of skin, liver, and mucosa exacerbated under steroid tapering and extracorporal photopheresis was initiated. Control of GvHD was unsatisfying, even under this combination, and ruxolitinib was added. Signs of GvHD regressed completely under this combination, steroids were terminated, and intervals of photopheresis could be extended. The patient is well and alive without active GvHD at day +565 after alloSCT (Karnofski score 90%) (Table 1).

In summary, the response of GvHD to supplementation of GvHD-therapy with ruxolitinib was excellent in patients without active gut involvement (n=4, CR=3, PR=1). There were no long-term hematological as well as nonhematological toxicity; especially there were no opportunistic infections. In four patients with uncontrolled acute grade IV GvHD and severe diarrhea (#2, #4, and #5, #8), no response was seen (NC=4) (Table 2).

## 4. Discussion

Therapy of Graft-versus-Host disease with ruxolitinib in conjunction with other drugs led to a response rate of 50% (4/8) in this investigation. Three remissions were complete and one partial, respectively. Generally, these results are in accordance with those published by other investigators. Zeiser et al. reported in their landmark paper data from 95 patients with steroid-refractory acute (n=54) and chronic (n=41) GvHD and described for both groups a response rate above 80% [5]. Other investigators published data from 8 to 13 patients: Maldonado et al. described in eight younger patients with acute and chronic GvHD a response rate of 100% (CR n=3, PR n=5) and Assouan et al. were successful with a partial or complete remission in 7/10 patients [9, 10]. Another group described use of ruxolitinib for GvHD treatment in paediatric patients suffering from acute GvHD [11]. Thirteen patients suffering from acute grade II-IV GvHD were treated with ruxolitinib. Here, the overall response rate was 38% (5/13).

The published data suggest that ruxolitinib is active against acute and chronic GvHD, independently, from the affected organ. Even for pulmonary and intestinal GvHD positive effects have been described [5, 12]. However, valid response data for different organ systems to ruxolitinib are unavailable, so far. The oral administration with a presumably poor resorption of the drug seems to be a problem in patients with severe diarrhea, even when the pills are finely grounded. In the present investigation, GvHD in four patients with intestinal disease and high-volume diarrhea did not respond to ruxolitinib. Comparable poor results in intestinal GvHD have been described by Khandelwal et al. [11]. Alternatively, since even primary therapy of intestinal GvHD is often a major challenge, an intrinsic resistance could be postulated [13]. However, Zeiser et al. reported successful treatment of intestinal GvHD with ruxolitinib and proved their results histologically [5]. Mori et al. were successful with ruxolitinib in conjunction with ATG in a case of stage 4 intestinal GvHD [14]. These controversial results need further investigation. A parenteral formulation of ruxolitinib could be helpful. The available data do not allow defining a position of ruxolitinib in therapeutic algorithm of acute or chronic GvHD so far. The course from patient #1 supports the evidence for an anti-GvHD activity of ruxolitinib monotherapy; however, all investigators have used it in conjunction with other drugs in higher treatment lines so far.

Furthermore, our report shows that ruxolitinib is not only an option in steroid-refractory GvHD; it can be used as a substitute for other drugs like CNI in aHUS after alloSCT or to spare steroids [15]. Since some authors postulate evidence for an effectivity against GvHD effect while the GvL-effect of the allogeneic graft seems to be preserved, it might be an interesting option in GvHD prophylaxis especially in high-risk patients [16, 17]. There were no long-term toxicity in our responding patients, but we have to admit that organ toxicity is difficult to evaluate in these patients since it could also be a sign of GvHD. This issue needs further systematic investigation in prospective trials.

The optimal dose of ruxolitinib and the length of therapy need further research. The definite position of ruxolitinib in prophylaxis and treatment of GvHD has to be clarified by prospective trials [18, 19].

## Figures and Tables

**Table 1:** Patient's details. SLL: small lymphocytic lymphoma, AML: acute myeloid leukaemia, MM: multiple myeloma, OMF: osteomyelofibrosis, CLL: chronic lymphatic leukaemia, FL: follicular lymphoma, Mud: matched unrelated donor, Mrd: matched related donor, D: day after alloSCT, MMF: mycophenolate mofetil, CSP: cyclosporine A, MSC: mesenchymal stem cells, ECP: extracorporal photopheresis, CR: complete remission, PR: partial remission, NC: no change. (1) CNI-replacement; (2) steroid sparing; 4^th^/5^th^: therapy line.

Pat.	Gender/age	Diagnosis	TX-type, HLA-match	Onset of GvHD	GvHD (grade)/involved Organs	Special complications	Therapy of GvHD Therapy line, Indication for R.	Outcome	Immunosuppression at follow-up	Follow-up
#1	Male, 37	SLL, p53del	Mud, 10/10	D +31	Acute (III) and chronic (ext.), gut, liver, skin, Overlap-syndrome	CNI associated aHUS	Steroids, MMF, Ruxolitinib ^1, 2^	CR of GvHD, KI 90%	Ruxolitinib (on taper)	D +804

#2	Male, 62	AML FLT3-ITD+	Mud, 10/10	D +71	Acute (IV), gut, liver, steroid-refractory	None	CSP, steroids, MMF, Ruxolitinib (4^th^), MSC	NC, Death from refractory GvHD	n. a.	D +154

#3	Female, 59	MM	Mrd (identical sibling)	D +164	Chronic (ext.), Lung, skin, liver	CNI associated aHUS	Steroids, MMF, Ruxolitinib ^1, 2^	PR, cGvHD improved, death from infection	n. a.	D +768

#4	Male, 68	OMF	Mud, 10/10	D +141	Acute (IV), gut, skin	None	CSP, steroids, MMF, Ruxolitinib (4^th^)	NC, Death from refractory GvHD	n. a.	D +209

#5	Male, 64	AML	Mud, 10/10	D +98	Acute (IV), gut, skin	None	CSP, steroids, MMF, ECP, Ruxolitinib (5^th^)	NC, Death from refractory GvHD	n. a.	D +133

#6	Male, 36	CLL, p53del	Mud, 10/10	D +224	Acute (III) and chronic (ext.), liver, gut, skin, lung, Overlap-syndrome	Aspergillus pneumonia	CSP, steroids, MMF, ECP, Ruxolitinib ^1, 2^	CR, KI70%, cGvHD inactive	Ruxolitinib, MMF	D +1393

#7	Male, 63	FL	Mud, 10/10	D +30	Acute (III) and chronic (ext.), gut, skin, mucosa Overlap syndrome	None	CSP, steroids, MMF, ECP, Ruxolitinib (5^th^), ^2^	CR, GvHD in complete remission	CsA, Ruxolitinib, MMF, ECP (on taper)	D +565

#8	Male, 68	AML	Mud, 10/10	D +75	Acute (IV), gut, skin	None	CSP, steroids, MMF, Ruxolitinib (4^th^)	NC, Death from refractory GvHD	n. a.	D +148

**Table 2:** GvHD at ruxolitinib initiation, indication for ruxolitinib, diarrhea, and outcome of GvHD. CNI: calcineurin-inhibitor.

Patient	GvHD-control at initiation of ruxolitinib	Indication for ruxolitinib	Diarrhea at ruxolitinib initiation	Response of GvHD to ruxolitinib
#1	Controlled	(i) CNI-replacement (ii) Steroid sparing	None	Yes
#2	Uncontrolled	(i) Uncontrolled GvHD	Severe	No
#3	Partially controlled	(i) CNI-replacement (ii) Steroid-sparing	None	Yes
#4	Uncontrolled	(i) Uncontrolled GvHD	Severe	No
#5	Uncontrolled	(i) Uncontrolled GvHD	Severe	No
#6	Partially controlled	(i) Steroid sparing	None	Yes
#7	Partially controlled	(i) Steroid sparing	None	Yes
#8	Uncontrolled	(i) Uncontrolled GvHD	Severe	No

## Data Availability

Upon request we are able to provide all research data underlying this manuscript.
